# Plasma cell leukemia with soft tissue involvement; reporting a rare case

**DOI:** 10.1016/j.lrr.2024.100411

**Published:** 2024-01-09

**Authors:** Ahmed Bendari, Rahaf M. Abu Khalaf, Sunder Sham, Reham Al-Refai, Oana Vele, Alyssa Yurovitsky

**Affiliations:** a Department of Pathology and Laboratory Medicine, Lenox Hill Hospital, Northwell Health, NY, USA; b Faculty of medicine, Al-Quds, Palestine

**Keywords:** Plasma cell neoplasm, CD 138, Atypical plasmacytoid cells

## Abstract

Plasma cell leukemia (PCL) is a rare aggressive variant of multiple myeloma. PCL is diagnosed when clonal plasma cells constitute more than 20 % of the total circulating leukocytes or when the absolute plasma cell count exceeds 2 × 10^9^ /L. Extramedullary involvement including cavity effusion is frequently seen at the time of diagnosis. However, soft tissue involvement is rarely encountered with only one published case in the English literature. We report a 74-year-old man, who presented with progressive shortness of breath over a few months. Laboratory studies showed leukocytosis (32 × 109 /L) with 26 % peripheral plasmacytoid cells and significantly elevated lactate dehydrogenase (> 2500 U/L). Serum protein electrophoresis detected a monoclonal IgG lambda band. A 7.4 cm left hilar mass, bilateral pleural effusion, and multiple fluorodeoxyglucose (FDG)-avid subcutaneous nodules in the pelvic and gluteal regions were demonstrated on imaging. Gluteal nodule biopsy revealed diffuse infiltrative CD138+ and MUM1+ cells with aberrant CD4, CD30, and BCL2 expression. The Ki-67 proliferation index was 70 %. Bone marrow biopsy showed sheets of atypical plasma cells with lambda-restriction and CD138 and MUM1 expression without cyclin D1 and CD20 expression. These cells comprise approximately 70–80 % of the bone marrow cellularity. A similar immunophenotype was demonstrated in peripheral and bone marrow flow cytometry. Molecular and cytogenetics showed an abnormal clone with a complex karyotype including monosomy 13 and 14q deletion. Overall, these findings are consistent with a plasma cell neoplasm. Our case study illustrates soft tissue involvement in PCL, which is rarely seen.

## 1 Introduction

Plasma Cell Leukemia (PCL), an uncommon and aggressive subtype of plasma cell proliferative disorders, accounts for 0.5–3 % of plasma cell disorders [1]. Traditionally defined by an absolute plasma cell count exceeding 2 × 10^9^ /L or a plasma cell percentage surpassing 20 % in circulating cells [2,3], recent recommendations from the International Myeloma Working Group (IMWG) and WHO propose that meeting either criterion suffices for a PCL diagnosis [3]. Additionally, emerging research indicates that PCL may be identified at lower thresholds, with greater than 5 % circulating plasma cells [4,5]. Further categorization distinguishes primary PCL from secondary PCL. Primary PCL is occurring when the leukemic phase occurred de novo without prior Multiple Myeloma (MM) diagnosis, while, in secondary PCL, the leukemic phase arises within an established MM context [1,3]. Both primary and secondary PCL are very aggressive diseases with a poor prognosis. [2] This review presents a case of Primary PCL with soft tissue involvement.

## 2 Case presentation

**Fig. 1:**
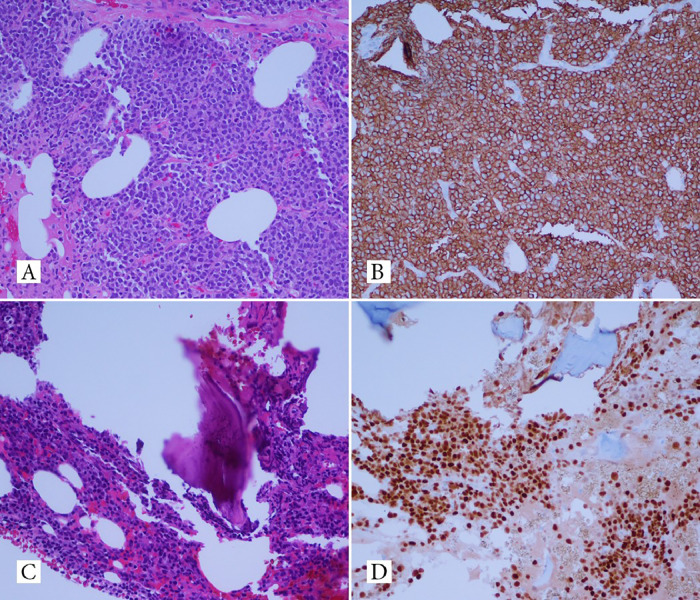
Gluteal biopsy shows diffuse infiltrate of neoplastic cells (A) that expressed CD138 (B). Bone marrow biopsy reveals sheets of atypical plasma cells comprising approximately 70–80 % of the cellularity (C) that expressed CD138 (Figure1-D).

We present a 74-year-old man who presented with progressive shortness of breath for three weeks. The patient had an unremarkable past medical history and did not smoke. Complete blood count was significant for anemia (Hb: 11 g/dL, Hct: 31.8 %) and leukocytosis (WBC: 32 × 109 /L, plasmacytoid cells: 26 %). The complete metabolic panel demonstrated elevated BUN, creatinine, AST, and significantly elevated LDH (> 2500 U/L). Peripheral blood and pleural fluid smears stained with Wright Giemsa stain showed numerous atypical plasmacytoid cells. M-spike (4.4 g/dL) and IgG lambda monoclonal band were detected on serum protein electrophoresis. The chest CT scan showed a 8.5 cm left perihilar mass, a 4.4 cm paravertebral mass, and superior diaphragmatic lymphadenopathy with moderate bilateral pleural effusions. PET imaging revealed multiple (FDG)-avid subcutaneous nodules in the pelvic and gluteal regions. Biopsy of one of the nodules demonstrated a diffuse infiltrate of neoplastic cells (Fig.1-A) that expressed CD138 (Fig.1-B), MUM1 and displayed aberrant expression of CD4, CD30, and BCL2. The Ki-67 proliferation index was 70 %. Bone marrow biopsy demonstrated sheets of atypical plasma cells comprising approximately 70–80 % of the cellularity (Fig.1-C). On immunohistochemistry, CD138 (Fig.1-D) and MUM1 highlighted the lambda-positive plasma cells. The plasma cells did not express kappa light chains, cyclin D1, and CD20. Flow cytometry demonstrated a monoclonal plasma cell population that displayed aberrant expression of CD56, CD2, and CD4, consistent with a plasma cell neoplasm. The tumor cells in the pleural fluid and subcutaneous gluteal mass shared similar morphology and immune-phenotyping profile with the tumor cells infiltrating the bone marrow. Cytogenetic analysis showed loss of chromosome Y, gain of 1q, partial deletion of 11q, monosomy 13, and interstitial deletion of chromosomes 8p and 14q. On fluorescence in site hybridization, the tumor cells were positive for 1q21/CKS1B gain. The overall findings, including clinical, imaging, laboratory and pathology were consistent with plasma cell leukemia.

## 3 Discussion

PCL is a rare, aggressive form of plasma cell disorders. Although both MM and PCL are on the same spectrum of plasma cell disorders and share some of the same cell surface markers (CD38 and CD138) [2,3], PCL is considered more aggressive and exhibits distinctive clinical and biological features [2,3]. Clinically, PCL patients are diagnosed at a median age younger by a decade than MM patients [2,3]. PCL tends to present more with extramedullary involvement than MM such as the liver, spleen, body cavities (pleura, pericardium, and peritoneum), and spinal cord [3,4,6]. This could be explained by that the neoplastic cells express fewer adhesive molecules (CD56, LFA-1, LFA-3) [3], leading to extramedullary accumulation of tumor cells [5]. In addition, PCL presents less frequently with bone involvement than MM [2,6]. This parallels our patient, who presents with a 7.4 cm left hilar mass, bilateral pleural effusion, and multiple fluorodeoxyglucose (FDG)-avid subcutaneous nodules in the pelvis and gluteal region.

The diagnosis of PCL is based on laboratory parameters, with more than 20 % clonal plasma cells by differential count of the leukocytes or by counting more than 2 × 10^9 /L circulating clonal plasma cells. PCL is characterized by decreased hemoglobin levels, cytopenia, hypercalcemia, renal insufficiency, and an increase in Lactate dehydrogenase and *β*2-microglobulin [2,4,6]. Serum and urine protein electrophoresis with immunofixation should also be obtained to identify a monoclonal immunoglobulin, with the IgG subtype being the most common in PCL, followed by light chain only PCL. A bone marrow biopsy with cytogenetics should be performed on all patients diagnosed with PCL. Any soft tissue mass identified with imaging should be biopsied to evaluate possible extramedullary involvement. The most common characteristic cytogenetic finding in PCL is chromosome 14 translocation, followed by 13q deletion (whole/partial), 17p deletion, and 1q amplification. It has been observed that *TP53* and *DIS3* mutations are more common in PCL than MM [2].

The median overall survival has been reported to be less than 1 year and due to the aggressive nature of PCL, immediate disease control is warranted to prevent disease-related complications and early mortality. Patients with detectable circulating PCs by conventional blood count, even if less than 20 %, should be considered for treatment similar to PCL [3].

The management of PCL is based on limited clinical trials, retrospective studies, and expert consensus due to the rarity of the disease. The current treatment strategies include systemic therapy with chemotherapy and immunomodulatory drugs, stem cell transplantation, supportive care, and the management of disease-related complications. Chemotherapy regimens commonly used in the treatment of PCL include bortezomib-based combinations, such as VAD (vincristine, doxorubicin, and dexamethasone) or CyBorD (cyclophosphamide, bortezomib, and dexamethasone) [7]. Additional options may include the use of immunomodulatory drugs (IMiDs) such as lenalidomide or pomalidomide, often in combination with dexamethasone [8]. Proteasome inhibitors such as carfilzomib may also be considered in the treatment of PCL. The choice of regimen is based on the patient's overall health, disease status, and treatment goals, and may be individualized to each patient. Stem cell transplantation, particularly autologous stem cell transplantation (ASCT), may be considered for eligible patients with PCL [9]. ASCT has shown promise in extending survival in PCL patients, especially those who have achieved a partial response or better with induction therapy. Allogeneic stem cell transplantation may also be considered in certain cases, particularly in younger, fit patients with high-risk disease, and the availability of a suitable donor [8]. Supportive care plays a crucial role in the management of PCL. This includes the management of disease-related complications such as anemia, hypercalcemia, renal dysfunction, and bone disease [10]. Patients may require treatment with bisphosphonates, erythropoietin, or transfusions to address specific symptoms and complications related to their disease. Additionally, managing treatment-related toxicities, assessing, and addressing patients' psychosocial needs, and considering palliative care interventions are integral components of supportive care in PCL [7].

## 4 Conclusion

In conclusion, this case report highlights the rare occurrence of soft tissue involvement in plasma cell leukemia (PCL), as well as the aggressive nature of the disease. The patient presented with extramedullary manifestations, including a hilar mass, pleural effusion, and subcutaneous nodules, demonstrating the diverse clinical manifestations of PCL. The extensive immunophenotyping and molecular cytogenetic analysis provided valuable insights into the diagnosis and characterization of the disease. This case underscores the importance of considering PCL as a differential diagnosis in patients presenting with unusual soft tissue involvement, and the need for further research to better understand and effectively manage this rare variant of multiple myeloma.

## Consent to participate/ informed consent

Written informed consent was obtained from the patient for her data to be used anonymously for teaching and publications.

## Ethical approval

Not applicable.

## Funding

Not applicable.

## CRediT authorship contribution statement

**Ahmed Bendari:** Conceptualization, Data curation, Formal analysis, Investigation, Validation, Visualization, Writing – original draft, Writing – review & editing. **Rahaf M. Abu Khalaf:** Conceptualization, Data curation, Writing – original draft, Writing – review & editing. **Sunder Sham:** Writing – original draft, Writing – review & editing. **Reham Al-Refai:** Writing – original draft, Writing – review & editing. **Oana Vele:** Writing – original draft, Writing – review & editing. **Alyssa Yurovitsky:** Conceptualization, Validation, Writing – original draft, Writing – review & editing.

## Declaration of competing interest

The authors declare no conflicts of interest.
